# Draft Genome Sequence of *Arthrobacter crystallopoietes* Strain BAB-32, Revealing Genes for Bioremediation

**DOI:** 10.1128/genomeA.00452-13

**Published:** 2013-07-05

**Authors:** M. N. Joshi, A. S. Pandit, A. Sharma, R. V. Pandya, S. M. Desai, A. K. Saxena, S. B. Bagatharia

**Affiliations:** Gujarat State Biotechnology Mission, Department of Science & Technology, Udyog Bhavan, Gandhinagar, Gujarat, India

## Abstract

*Arthrobacter crystallopoietes* strain BAB-32, a Gram-positive obligate aerobic actinobacterium having potential application in bioremediation and bioreduction of a few metals, was isolated from rhizosphere soil of Gandhinagar, Gujarat, India. The draft genome (4.3 Mb) of the strain revealed a few vital gene clusters involved in the metabolism of aromatic compounds, zinc, and sulfur.

## GENOME ANNOUNCEMENT

The genus *Arthrobacter* was established within the family *Micrococcaceae* by Conn and Dimmick in 1947 (1). These bacteria are sporogenous, coryneform, rod-shaped actinomycetes having typically high G+C content (59 to 66%) and A-type (A3a or A4a) peptidoglycans with l-lysine as the dibasic amino acid (2). Since the discovery of this genus, over 52 species having pleomorphism and erratic Gram natures (Gram negative to Gram positive) have been placed in the genus. These species have been isolated from distinct resources such as soil (3), cheese (4), clinical specimens (5), paintings (6), seals (7), alpine ice caves (8), fish (9), wastewater reservoir sediment (10), air (11), and ocean sediments (12).

Previous studies revealed that species in the genus *Arthrobacter* have the capacity to degrade various substrates, including complex hydrocarbons, herbicides, and pesticides (13), and a few species can be further exploited for industrial application due to their production of various enzymes (14). Due to bioremediation abilities and applications in various industries, members of the genus *Arthrobacter* are of interest for genomic research. Therefore, *Arthrobacter crystallopoietes* strain BAB-32 was subjected to whole-genome sequencing. The bacterium was isolated from rhizospheric soil of *Syzygium cumini* (23°14′12.1″N, 72°40′37.5″E, Gandhinagar, Gujarat, India) by using the serial dilution method. 

Genome sequencing of the strain was done using the Ion Torrent personal genome machine with the Ion Torrent Server (Torrent suite version 3.2). Total data of 1,538,164 mate-paired reads with 30× coverage (mean read length 104 bp with the highest read of 201 bp) were obtained. *De novo* assembly was performed using the MIRA-3 assembler (v3.1.0). The annotation of the genome was performed using the NCBI Prokaryotic Genomes Automatic Annotation Pipeline (PGAAP) (http://www.ncbi.nlm.nih.gov/genomes/static/Pipeline.html) and the RAST server utilizing GeneMark, Glimmer, and tRNAscan-SE tools (15) with the SEED database (16).

The total length of the genome was found to be 43,486,070 bp allocated into 347 contigs, each having a size of >500 bp (scaffold *N*_50_, largest contig 100,415 bp). The G+C content was 66.6%. The chromosome of the strain BAB-32 harbored 4,118 protein-coding genes, 51 transfer RNAs, and 3 ribosomal RNAs. The genome sequence of the species contained coding sequences for various metallic enzymes, like metal-dependent hydrolase, aromatic aminotransferase, Zn-dependent oxidoreductases, iron sulfur oxidoreductase subunit, metal sulfur cluster biosynthetic enzyme, Mg-chelatase subunit, quinone oxidoreductase, zinc metallopeptidase, zinc protease, Fur protein (for ferric uptake), uricase (urate oxidase), and phenyl acetic acid degradation protein, providing the scope of the utilization of this strain in the bioreduction of various contaminants from the environment. Antibiotic coding genes, such as antibiotic biosynthesis monooxygenase, putative multidrug resistance efflux pump, bacteriocin, bleomycin resistance, and penicillin tolerance protein genes, are also present in the genome, which may have industrial and medical applications. Therefore, the availability of the draft genome of the species will assist in future functional and comparative analyses of the genome.

### Nucleotide sequence accession number.

The draft genome sequence of *Arthrobacter crystallopoietes* BAB-32 has been deposited at GenBank under accession number ANPE00000000.2.
